# Evolutionary conflicts and adverse effects of antiviral factors

**DOI:** 10.7554/eLife.65243

**Published:** 2021-01-15

**Authors:** Daniel Sauter, Frank Kirchhoff

**Affiliations:** 1 Institute of Molecular Virology, Ulm University Medical CenterUlmGermany; 2 Institute of Medical Virology and Epidemiology of Viral Diseases, University Hospital TübingenTübingenGermany; University of GenevaSwitzerland; University of GenevaSwitzerland

**Keywords:** restriction factors, viral pathogens, adverse effects, innate immunitya

## Abstract

Human cells are equipped with a plethora of antiviral proteins protecting them against invading viral pathogens. In contrast to apoptotic or pyroptotic cell death, which serves as ultima ratio to combat viral infections, these cell-intrinsic restriction factors may prevent or at least slow down viral spread while allowing the host cell to survive. Nevertheless, their antiviral activity may also have detrimental effects on the host. While the molecular mechanisms underlying the antiviral activity of restriction factors are frequently well investigated, potential undesired effects of their antiviral functions on the host cell are hardly explored. With a focus on antiretroviral proteins, we summarize in this review how individual restriction factors may exert adverse effects as trade-off for efficient defense against attacking pathogens.

## Introduction

**Figure 1.**
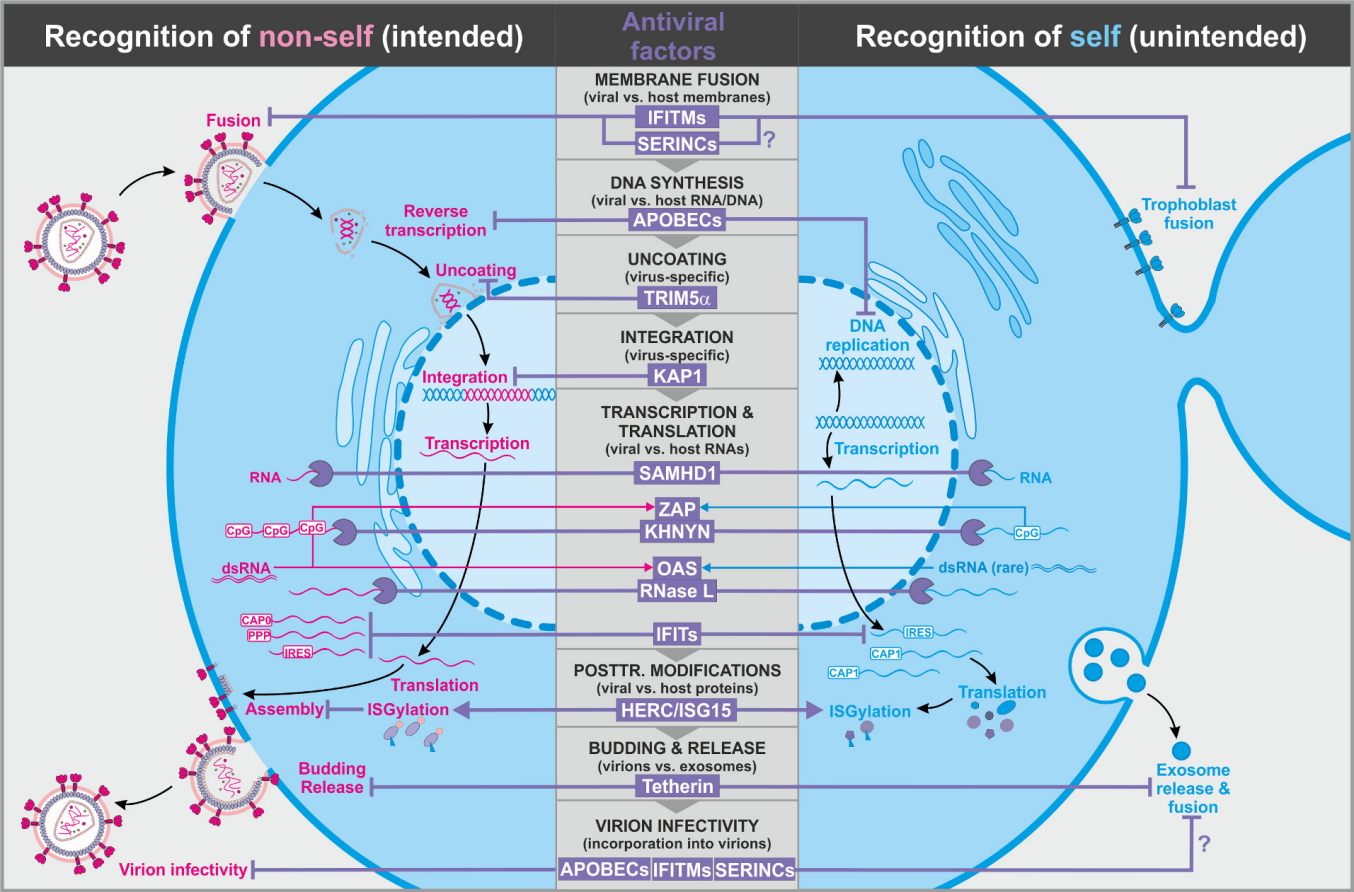
Antiviral factors targeting components of the virus. The retroviral replication is exemplarily shown to illustrate antiviral host factors (violet) that directly target viral proteins, nucleic acids, and membranes during essentially all steps of the viral life cycle. While some factors successfully distinguish between self (blue, right panel) and non-self (pink, left panel), others may have unintended side effects on the host as they also target cellular factors. CpG: cytosine guanine dinucleotides; dsRNA: double-stranded ribonucleic acid; CAP0: 5′ mRNA cap with unmethylated ribose hydroxy-groups; CAP1: 5′ mRNA cap with methylated ribose hydroxy-group; IRES: internal ribosome entry site; PPP: 5′-triphosphate group without cap; abbreviations of protein names are explained in the text.

**Figure 2.**
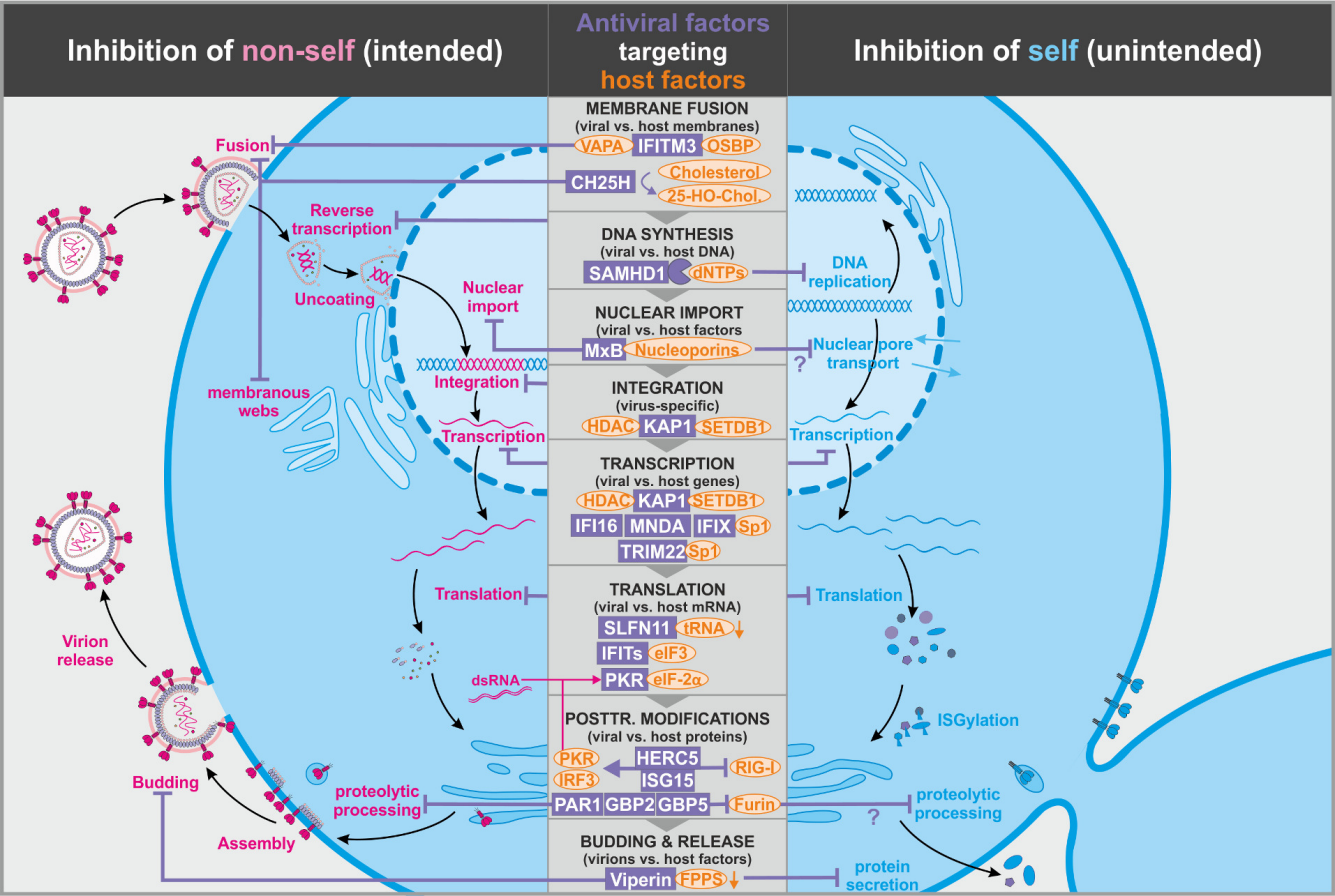
Antiviral factors modulating virus-dependency factors. Several antiviral host proteins (violet) suppress viral replication (left panel) by modulating the stability, localization, or activity of cellular factors (orange) involved in the viral replication cycle. Since these host factors also play important roles in the cell, their inhibition may be associated with detrimental side effects (right panel). dsRNA: double-stranded ribonucleic acid; tRNA: transfer ribonucleic acid; 25-HO-Chol.: 25-hydroxy-cholesterol; abbreviations of protein names are explained in the text.

Restriction factors are structurally and functionally highly diverse cellular proteins that represent important effectors of the early immune response and may target viral pathogens by numerous mechanisms at essentially every step of their replication cycle (Ghimire et al., 2018; Harris et al., 2012; Kluge et al., 2015; Malim and Bieniasz, 2012). The term ‘restriction factor’ has already been established about 50 years ago following the discovery that the Friend virus susceptibility protein 1 (Fv1) protects mice against otherwise lethal Murine leukemia virus (MLV) infections (Lilly, 1970). Since then, many cellular factors have been reported to exert antiviral activity. Among the first to be molecularly characterized was MxA, which protects cells against viruses replicating in the nucleus, such as influenza A virus (IAV) (Staeheli et al., 1986). It is debated which of the many antiviral factors that have been reported deserve the designation restriction factor. Proteins that are not directly involved in antiviral immunity may still suppress viral replication if they modulate cellular pathways that are exploited by viruses. Thus, antiviral activity, particularly in overexpression settings, is insufficient for definitive assignment, and there is no unambiguous definition of restriction factors (Doyle et al., 2015; Harris et al., 2012; Kluge et al., 2015). While exceptions do exist, most of these cellular antiviral factors share a few common characteristics. Although they are constitutively expressed in many cell types to provide immediate protection against viral pathogens, most of them are further upregulated by interferons (IFNs) upon sensing of viral invaders (Doyle et al., 2015; Harris et al., 2012; Kluge et al., 2015). Innate antiviral factors have the task to protect us against a large variety of viruses. To fulfill this task, many restriction factors directly target evolutionarily conserved structural features (e.g. viral genomes) or events in the viral replication cycle (e.g. fusion, budding) and exert broad antiviral activity (Table 1, Figure 1; Kluge et al., 2015). In contrast, some restriction factors inhibit viral pathogens more indirectly by limiting the availability of cellular resources such as nucleotides, transcription factors, or other virus-dependency factors (Braun et al., 2019; Hotter et al., 2019; Hrecka et al., 2011; Krapp et al., 2016; Laguette et al., 2011; Table 2, Figure 2).

**Table 1.** Selection of antiviral factors directly targeting viral replication (abbreviations are explained in the text).

Antiviral factor(s)	Target(s)	Discrimination between self and non-self	Effect on viral replication	(Potential) Unwanted effects on host cell	
				Immediate	Long term
IFITMs	Fusing membranes	Membrane curvature, lipid composition	Impaired fusion of viral and host membranes	Impaired fusion of cellular membranes	Constraints in membrane fusion (e.g. Syncytin-mediated trophoblast fusion)
SERINCs	Fusing membranes	Not known (viral glycoprotein dependency?)	Impaired fusion of viral and host membranes	None (?)	
TRIM5α, Fv1	Retroviral capsids	Specific protein-binding	Untimely uncoating	None (?)	Constraints in the co-option of endogenous retroviral capsid proteins
KAP1	Retroviral integrase	Specific protein-binding	Inhibition of integration	None	
ZAP/TRIM25/ KHNYN	RNA	CpG content	Degradation of viral RNA	Degradation of host RNA	CpG depletion (?)
RNAse L	RNA	dsRNA-dependent, OAS-mediated activation	Degradation of viral RNA	Degradation of host RNA	Avoidance of dsRNA
SAMHD1	RNA	Not known	Degradation of viral RNA	Degradation of host RNA (?)	
IFITs	RNA	IRES, modification of 5′ RNA ends (cap-1 vs. cap-0)	Inhibition of viral translation	Inhibition of cellular translation (?)	Depletion of IRES structures, constraints in mRNA capping
HERC5/ISG15	Numerous viral proteins (e.g. HIV-1 Gag, HPV capsid)	Preferred ISGylation of newly translated proteins	Inhibition of viral protein function	Inhibition of host protein function	
Tetherin	Budding membranes	Localization in lipid rafts	Inhibition of virion release	Inhibition of exosome release, inhibition of cell division (?)	
APOBECs	ssDNA, RNA	Partially sequence dependent	Introduction of lethal hypermutations in the viral genome	Emergence of detrimental mutations	Depletion of specific dinucleotides

**Table 2.** Selection of antiviral factors indirectly targeting viral replication (abbreviations are explained in the text).

Antiviral factor(s)	Target(s)	Discrimination between self and non-self	Effect on viral replication	(Potential) Unwanted effects on host cell	
				Immediate	Long term
IFITM3	VAPA, OSBP	Membrane curvature, lipid composition	Impaired fusion of viral and host membranes	Impaired fusion of cellular membranes	Constraints in membrane fusion (e.g. syncytin-mediated trophoblast fusion)
CH25H	Cholesterol	Not known	Impaired fusion of viral and host membranes, impaired membraneous web formation	Impaired fusion of host membranes (?)	
SAMHD1	dNTPs	Not known	Limits reverse transcription/viral DNA replication	Inhibition of host DNA replication	Regulation of SAMHD1 activity in dividing cells
MxB	Nucleoporins	Simultaneous interaction with viral (capsid) proteins	Reduced nuclear import of subviral complexes	Impaired nuclear pore transport	Evolution of diverse nuclear pore variants
KAP1	NuRD complex/HDACs, SETDB1, transcription factors	Not known	Suppression of viral gene transcription, latency	Suppression of host gene transcription	
TRIM22	Sp1	Not known	Reduced Sp1-driven expression of viral genes	Reduced Sp1-driven expression of host genes	Constraints in Sp1-driven gene expression
IFI16, MNDA, IFIX	Sp1	Chromatinization status of the DNA	Reduced Sp1-driven expression of viral genes	Reduced Sp1-driven expression of host genes	Constraints in Sp1-driven gene expression
PKR	eIF-2α	Activation by dsRNA	Reduced translation of viral mRNA	Reduced translation of host mRNA	Avoidance of dsRNA
IFITs	eIF3	IRES, modification of 5′ RNA ends	Inhibition of translation	Inhibition of translation (?)	Depletion of IRES structures, mRNA capping (methylated)
SLFN11	tRNA	preferred targeting of tRNAs exploited by viruses	Reduced translation of viral mRNA	Reduced translation of cellular mRNA	Specific codon usage pattern
PAR1, GBP2, GBP5	Furin	Not known	Impaired furin-mediated maturation of viral (glyco)proteins	Impaired proteolytic activation of host proteins	Constraints in furin-mediated protein cleavage
HERC5/ISG15	Numerous host proteins (e.g. IRF3, RIG-I, PKR)	Preferred ISGylation of newly translated proteins	Several proposed inhibitory mechanisms	Modulation of host protein stability and function	
Viperin	FPPS, CTP	Not known	Inhibition of viral budding, inhibition of viral RNA polymerization	Inhibition of cellular protein secretion and potentially cellular RNA synthesis	

Due to their rapid replication rates, enormous number of progeny, and frequently high mutation rates, many viruses quickly adapt to their respective host environments. Altogether, viruses have evolved sophisticated strategies to evade or directly counteract many restriction factors. For example, they frequently mimic the properties of their host cells to avoid recognition by the cell. In addition, viral pathogens may capture cellular genes and transform them into effective tools against antiviral defense mechanisms (Duggal and Emerman, 2012; Nchioua et al., 2020b; Sauter and Kirchhoff, 2018). This not only allows them to exploit host factors for their own purposes, but the cellular origin also makes it even more difficult for the host to discriminate between self and non-self. As a consequence of the need to maintain activity against evolving pathogens or to provide protection against newly emerging viruses, many restriction factors evolve particularly fast and show evolutionary signatures of adaptation (Cagliani et al., 2014; Duggal and Emerman, 2012; Pyndiah et al., 2015). Particularly regions in antiviral proteins that directly interact with viral components either to inhibit or be targeted by them for counteraction show strong evidence for positive selection. One important consequence of this ever-ongoing virus–host arms race is that restriction factors are usually highly effective against poorly adapted viruses from other species thereby frequently representing potent barriers to successful cross-species transmissions. In contrast, they are often hardly effective against well-adapted viral pathogens in their natural hosts. Notably, their ability to interact with viral components allows some restriction factors to not only directly restrict viral pathogens but also act as pattern recognition receptors that induce and boost antiviral immune responses (Galão et al., 2012; Hotter et al., 2013; Jakobsen et al., 2013; Jønsson et al., 2017).

One formidable challenge for the host is the evolution of antiviral factors that effectively protect against foreign viral invaders without harming the cell. While it is advantageous for adaptive immune mechanisms to be specific for individual invading pathogens, innate immunity must provide broad-based protection against a huge variety of diverse potential viral invaders. This includes viruses that the individual or even the entire host species has never encountered before. Thus, it is obvious that innate immune factors need to strike a fine balance between protection against a broad range of viral pathogens and limiting the risk of unwanted off-target effects on the host organism. Effective antiviral defense mechanisms might cause undesired adverse effects by numerous mechanisms, for example because the antiviral factors do not perfectly distinguish between self and foreign, or because virus-dependency factors that are depleted are also important for cellular functions. In addition, immune activation alters the concentrations and activities of several cellular factors, many of which also fulfill important physiological functions. Finally, multiple cellular resources and machineries are redirected for defense or shutdown, so that they cannot perform their regular functions anymore. Altogether, it is evident that there are trade-offs between effective innate antiviral immune mechanisms and potential side effects on the host cell and consequently organism.

The molecular mechanisms of antiviral restriction factors and their viral antagonists have received substantial attention and have been the topic of several in-depth reviews (Harris et al., 2012; Malim and Bieniasz, 2012; Sauter and Kirchhoff, 2018). In contrast, adverse effects of the antiviral activities of host restriction factors have received little attention, although they may play important roles in the clinical outcomes of viral infections. To close this gap, we here discuss some of the potential side effects associated with antiviral host proteins. We focus on three ways, by which antiviral proteins may result in detrimental effects. First, restriction factors may fail to discriminate between self and non-self. This is not surprising given that viruses exploit the cellular protein synthesis and trafficking machineries and all viral components are ultimately derived from the host cell. Second, some restriction factors not only suppress viral replication but also perform other functions in the cell. Consequently, their induction in response to infection or their counteraction by viral antagonists may perturb their physiological activity and thus, the state or function of the cell. Third, several antiviral factors do not target the pathogen directly but generate an antiviral environment by limiting the availability of so-called virus-dependency factors. These host factors are required for viral replication but generally also involved in cellular processes. A better understanding of trade-offs associated with the emergence of innate immunity factors is important because (1) side effects of antiviral proteins may contribute to the pathogenesis of infectious diseases, particularly in chronic viral infections, (2) aberrant expression and/or activity of antiviral proteins may result in disorders such as inflammatory auto-immune diseases, and (3) therapeutic approaches exploiting host restriction factors need to consider potential adverse effects. The detrimental effects of aberrant chronic immune activation in chronic viral infections, such as HIV/AIDS, are well documented (Bloch et al., 2020; Deeks, 2011). Accumulating evidence suggests that severe coronavirus disease 2019 (COVID-19) is also driven by excessive immune activation and expression of pro-inflammatory cytokines (the so-called ‘cytokine storm’) in response to SARS-CoV-2 infection (Lariccia et al., 2020; Quirch et al., 2020). The focus of the present review is on side effects of specific cell-intrinsic antiviral effectors. Our aim is not only to illustrate evolutionary conflicts associated with the acquisition of cellular antiviral proteins but also to provide insights into their physiological roles and potential adverse effects in virally infected cells and the host organism in general. Due to the constantly increasing number of newly discovered cellular proteins with antiviral activity, we had to limit our review to the description of a few exemplary factors. Since many of them are best characterized for their effects on HIV-1, we focus on antiretroviral proteins to illustrate different concepts of self versus non-self discrimination and mechanisms leading to unwanted side effects.

### Suppression of viral entry

In order to replicate, viral pathogens must deliver their genetic material into the host cell. Preventing entry of enveloped viruses is advantageous for the host because it minimizes potentially harmful interactions with the pathogen and avoids manipulation of the host cell by intracellular viral factors. Individual cells may prevent entry of enveloped viruses by selfish or selfless mechanisms: Cells may exclusively protect themselves by downmodulating cellular receptors and cofactors required for infection or by expressing antiviral factors that inhibit fusion with viral particles. Alternatively, infected cells may prevent incorporation of functional viral envelope proteins in progeny virions or induce the incorporation of cellular factors that reduce viral infectiousness and, thus, protect bystander cells rather than themselves. All these modes of action are non-exclusive, and, as outlined below, some antiviral factors may act in both the viral target and producer cells.

Several cell-intrinsic entry inhibitors exert very broad antiviral activity. For example, members of the IFN-induced transmembrane (IFITM) family have been reported to protect cells against a large variety of viral pathogens (e.g. retro-, orthomyxo-, flavi-, rhabdo-, influenza A, and coronaviruses) (Bailey et al., 2014; Diamond and Farzan, 2013; Shi et al., 2017; Smith et al., 2014; Figure 1, left). At least three of the five human IFITM proteins (i.e. 1, 2, and 3) exert antiviral activity. IFITM3 has been suggested to exert its antiviral activity by interfering with the homeostasis of intracellular cholesterol levels. More specifically, IFITM3 induces the intracellular accumulation of cholesterol by interacting with the cholesterol regulatory factor oxysterol-binding protein (OSBP) and vesicle-membrane-protein-associated protein A (VAPA) (Amini-Bavil-Olyaee et al., 2013; Figure 2). As a result, fusion of vesicular stomatitis virus (VSV) particles and potentially other viruses with the host cell membrane is inhibited. While the molecular mechanisms of IFITM1 and IFITM2 remain less clear, these two factors have also been suggested to restrict viral entry by modulating membrane fluidity and curvature (Li et al., 2013). It is well known that the lipid composition of purified virions differs from that of typical mammalian cells (Ivanova et al., 2015) and that the glycerophospholipid composition of membranes affects their curvature (Casares et al., 2019). Since viral particles are usually much smaller than cells, they require stronger membrane curvature. In addition, fusion of viral membranes with cellular membranes may require strong negative bending, and the compositions of the viral and target cell membranes play key roles in the initiation and efficiency of fusion and thus viral entry (Stiasny and Heinz, 2004, Alexandrov et al., 2013). Nevertheless, virus–host and host–host membrane fusion events share several overlapping characteristics, and the broad antiviral activity of IFITMs may come at the cost of altered host membrane fusion. For example, increased IFITM levels have recently been shown to inhibit trophoblast fusion, a critical step in placenta formation (Buchrieser et al., 2019; Figure 1, right). As a result, the syncytiotrophoblast does not form, and the fetus is restricted in growth. Like many other antiviral defense factors, IFITMs are strongly upregulated in the presence of IFNs. Thus, this undesired effect of IFITMs may explain why inflammation and IFNs are associated with premature termination of pregnancies and embryopathies (Yockey and Iwasaki, 2018).

Another antiviral host protein modulating membranes is the IFN-inducible cholesterol-25-hydroxylase (CH25H). This factor inhibits not only fusion during entry of a variety of enveloped viruses (e.g. HCV, VSV, HSV, HIV, EBOV, RVFV, SARS-CoV-2, and Nipah virus) (Zhao et al., 2020) but also HCV RNA replication by interfering with the formation of membranous webs that serve as HCV replication factories (Anggakusuma et al., 2015; Figure 2). Both of these inhibitory effects require the enzymatic activity of CH25H and are mediated by its product 25-hydroxy-cholesterol (25HC). This also illustrates that membrane-modulating factors such as CH25H may interfere with viral pathogens at several steps of their replication cycle. Whether CH25H and 25HC also interfere with physiological membrane fusion within or between cells remains to be determined.

While IFITM proteins and CH25H seem to mainly (but not exclusively) exert their effects in viral target cells (Compton et al., 2014), the antiviral factors SERINC3 and SERINC5 can be efficiently incorporated into virions and prevent subsequent rounds of infection, at least in the absence of an effective viral antagonist (Rosa et al., 2015; Usami et al., 2015). Although the exact inhibitory mechanism is unclear, it has been shown that SERINC5 prevents delivery of the viral core into target cells by impairing the fusion process (Buffalo et al., 2019; Sood et al., 2017; Figure 1, left). In the case of HIV-1, the effect of SERINCs also depends on the specific envelope glycoproteins and may involve changes in their clustering and/or conformation (Chen et al., 2020; Featherstone and Aiken, 2020). Thus, the presence of viral glycoproteins may help the cell to distinguish between cell–cell and virus–cell fusion events. The full antiviral spectrum of SERINC5 and its family members remains to be determined. Compared to IFITMs, it seems more confined to retroviruses (Heigele et al., 2016), although it has recently been reported that SERINC5 also suppresses the production of hepatitis B virus particles (Liu et al., 2020).

In contrast to IFITMs, CH25H and many other restriction factors, SERINC3 and 5 are not upregulated by IFN or other proinflammatory cytokines (Rosa et al., 2015). The physiological role of SERINCs is under debate. These proteins were named SERINCs because it has initially been suggested that they mediate SERine INCorporation into lipid membranes (Inuzuka et al., 2005). However, more recent data did not confirm effects of SERINC5 on the lipid composition of cells or viral particles (Trautz et al., 2017). Furthermore, SERINC5^−/−^ mice show no obvious phenotypic defects (Timilsina et al., 2020). Altogether, it has been established that SERINC expression levels do not change under inflammatory conditions, and recent data suggest that SERINC5 might not exert important functions beyond antiviral immune defense. Thus, SERINC5 may impair the infectivity of retroviral particles without causing detrimental side effects.

### Inhibition of viral reverse transcription and uncoating

Virion fusion with the cell membrane allows viral genomes to enter the cell. In the case of retroviruses, the viral RNA genome is reverse transcribed into linear double-stranded DNA and transported into the nucleus for integration into the host cell genome. Initially, it was thought that retroviral capsids rapidly disassemble upon cytosolic entry. However, recent data suggest that the HIV-1 capsid probably remains intact, or nearly so, until after nuclear import (Novikova et al., 2019). The integrity of the capsid structure is thought to be important for intracellular trafficking, suppression of innate immune sensing, reverse transcription, and nuclear import of the viral genome (James and Jacques, 2018; Le Sage et al., 2014). Thus, reverse transcription and uncoating are tightly linked and have to proceed in a well-coordinated manner for successful infection. One antiviral factor that perturbs this process is tripartite motif-containing protein 5α (TRIM5α). This protein belongs to a large family of ~100 TRIMs (Han et al., 2011), many of which are involved in the innate response to viral infection (Koepke et al., 2021). TRIM5α directly interacts with retroviral capsids and results in accelerated uncoating and consequently inhibition of reverse transcription (Ganser-Pornillos et al., 2011; Stremlau et al., 2004; Figure 1, left). The high specificity of TRIM5α–capsid interactions and the absence of capsid-like structures from most host cells minimizes the risk of unintended off-target effects but at the same time enables retroviral pathogens to develop resistance. In fact, the evolution of the interaction interface between TRIM5α and retroviral capsids provides a prime example for the arms race between innate defense factors and viral evasion mechanisms. TRIM5α shows strong signatures of positive selection (Kaiser et al., 2007; Sawyer et al., 2005), particularly in the interaction interface with retroviral capsids (McCarthy et al., 2015). Consequently, TRIM5α acts in a species-specific manner. For example, the HIV-1 capsid efficiently interacts and is restricted by TRIM5α from rhesus macaques but is largely resistant to human TRIM5α (Stremlau et al., 2004), possibly due to protective shielding by cyclophilin A (Kim et al., 2019). Because of this high specificity, it was thought that TRIM5α only restricts retroviruses. Recent findings, however, suggest that TRIM5α is also active against some flaviviruses (Chiramel et al., 2019). Whether or not TRIM5α exerts a relevant physiological function and whether its induction by IFNs may be associated with detrimental effects is largely unknown. It has been reported, however, that TRIM5α overexpression induces morphological changes in HEK293T cells that are suppressed by interaction with the heat shock protein 70 (Hsp70) (Hwang et al., 2010).

Another factor, SAM domain and HD domain-containing protein 1 (SAMHD1) suppresses reverse transcription of various retroviruses by creating a cellular environment that is not permissive for viral replication (Hrecka et al., 2011; Laguette et al., 2011). Specifically, SAMHD1 is an enzyme that removes the triphosphate from dNTPs, thereby depleting cells of the pool of dNTPs required for reverse transcription (Goldstone et al., 2011; Lahouassa et al., 2012; Powell et al., 2011; Figure 2, left). The levels of dNTPs as well as the activity of SAMHD1 vary substantially between various cell types, and SAMHD1 mainly restricts retroviral replication in cells that have relatively low levels of dNTPs to start with, that is non-dividing macrophages and resting T cells (Baldauf et al., 2012; Descours et al., 2012; Hrecka et al., 2011; Laguette et al., 2011). In contrast to other antiviral factors, the expression levels of SAMHD1 are not altered by immune activation. Instead, the enzymatic and antiviral activities of SAMHD1 are regulated by post-translational modifications, that is phosphorylation and acetylation (Cribier et al., 2013). Since dNTPs are critical for host DNA replication, their depletion by SAMHD1 will keep cells in a non-dividing state (Figure 2, right). However, cell division is a key mechanism for successful immune responses. Thus, efficient reduction of the dNTP pool by activated SAMHD1 is obviously only an option for antiviral defense in specific cell types because it would otherwise exert detrimental immune suppressive effects. In addition, it is known that mutations in SAMHD1 are associated with the Aicardi–Goutières syndrome, and recent studies suggest roles of SAMHD1 in double-stranded break repair, genomic stability, and potentially some types of cancer (Coggins et al., 2020). Altogether, accumulating evidence suggests that altered SAMHD1 activity due to activation by the innate immune response or inhibition by lentiviral antagonists, that is Vpx and Vpr (Fregoso et al., 2013; Hrecka et al., 2011; Laguette et al., 2011), may have significant adverse effects on the cell.

### Nuclear import

Before retroviral DNA can be integrated into host chromosomes, subviral complexes need to enter the nucleus via nuclear pore complexes. This step is inhibited by the IFN-inducible protein MxB (Goujon et al., 2013; Kane et al., 2013; Liu et al., 2013), which directly interacts with the retroviral capsid (Fricke et al., 2014) and several nucleoporins and nucleoporin-like proteins (Dicks et al., 2018; Figure 2, Table 2). The positioning of MxB at the nuclear pore complex (NPC) is mediated by a nuclear localization signal-like sequence in its N-terminus. This sequence stretch is absent from its paralog MxA, which inhibits diverse viral pathogens, but not retroviruses (Haller et al., 2015). Notably, the composition of NPCs varies considerably within and between different cells, and not all of them may be efficiently targeted by MxB (Dicks et al., 2018; Kane et al., 2018). Thus, MxB-mediated inhibition of the retroviral pre-integration complex depends on the cell type and the import pathway that is used by the virus. While there is emerging evidence for a dysregulation of nuclear pore transport by MxB, this restriction factor may achieve some specificity by simultaneously interacting with components of the retroviral core.

### Proviral integration and transcription

Integration of the linear retroviral dsDNA into the host genome is essential for efficient viral transcription and productive infection. This step is inhibited by KRAB-associated protein-1 (KAP1), also known as TRIM28, another member of the TRIM family (Allouch et al., 2011). KAP1 inhibits proviral integration by inducing deacetylation of the retroviral integrase via recruitment of a protein complex including histone deacetylases (HDACs) (Figures 1 and 2, left). More importantly, the recruitment of HDACs and the histone methylase SETDB1 by KAP1 also results in epigenetic changes that induce heterochromatinization, repress transcription, and may therefore also promote viral latency (Figure 2, left). For example, latency of the Kaposi's sarcoma-associated herpesvirus has been shown to be regulated by KAP1 (Chang et al., 2009). Furthermore, KAP1 also plays a key role in silencing transposable elements, including endogenous retroviruses (Tie et al., 2018). Recent evidence suggests that KAP interacts with a variety of cellular factors involved in DNA interaction and is recruited to actively transcribed polymerase II promoters (Kauzlaric et al., 2020). Thus, the repressive activity of KAP1 is not specifically directed against viral genes. In line with this, it has been reported that KAP1 also governs the expression of tumor-suppressor genes (Serra et al., 2014). In contrast to many other antiviral factors, KAP1 is not further inducible by IFNs, possibly because changes in its expression or activity upon viral infection may result in unwanted effects on the host cell (Figure 2, right).

Viral pathogens must exploit cellular machineries for efficient transcription of their own genes, and recent studies suggest that some IFN-inducible antiviral factors limit the availability of cellular transcription factors to inhibit viral pathogens. Initially, it has been reported that TRIM22 suppresses basal HIV-1 transcription as it inhibits binding of the transcription factor Sp1 to the HIV-1 LTR promoter via a poorly described mechanism (Turrini et al., 2019; Turrini et al., 2015; Figure 2, left). More recently, it has been shown that nuclear members of the human PYHIN family (i.e. IFIX/PYHIN1, IFI16, and MNDA) directly interact with Sp1 via their pyrin domains, thereby limiting the availability of Sp1 for HIV-1 transcription (Bosso et al., 2020; Hotter et al., 2019; Figure 2, left). Sp1 is critical for efficient expression of multiple pathogens, and it has been reported that IFI16 restricts retro-, herpes-, and papillomaviruses, possibly by several non-exclusive mechanisms (Gariano et al., 2012; Johnson et al., 2014; Lo Cigno et al., 2015). It has been suggested that IFI16 may cooperatively bind dsDNA in a length-dependent manner and cluster into protein filaments (Morrone et al., 2014). Assembly into filaments is mediated by conserved residues in the pyrin domain and required for high-affinity binding of DNA via the HIN domains. Nuclear PYHIN proteins, including IFI16, were proposed to distinguish self from foreign (Morrone et al., 2014; Stratmann et al., 2015) by associating only with under-chromatinized foreign DNAs. However, the HIN domains of human PYHIN proteins known to be required for DNA interaction were dispensable for their antiretroviral activity (Bosso et al., 2020; Hotter et al., 2019). Instead, the pyrin domain of human PYHIN proteins competed with Sp1 binding sites in DNAs for Sp1 interaction. Sp1 is also involved in the expression of numerous cellular proteins that play roles in cancer and inflammatory diseases (Li and Davie, 2010; O'Connor et al., 2016; Safe et al., 2014). Thus, attenuation of Sp1 function by TRIM22 or PYHIN proteins will most likely also significantly reduce Sp1-driven expression of cellular genes and presumably affect multiple physiological and pathological processes (Figure 2, right).

### mRNA degradation and inhibition of viral mRNA translation

Although viral pathogens exploit the cellular protein synthesis machinery, a few characteristics (e.g. codon usage, CpG dinucleotide content, 5′ cap, formation of double strands, and/or specific secondary structures) may distinguish cellular from viral mRNAs. These characteristics are exploited by antiviral host factors such as ZAP, SLFN11, PKR, or IFITs to preferentially target viral transcripts (Nchioua et al., 2020a). However, many viruses mimic the mRNA structure and composition of their respective host species to evade restriction. For example, mammalian genomes show marked suppression of CpG dinucleotides, and it is long known that many RNA viruses mimic this feature of their vertebrate hosts (Cooper and Gerber-Huber, 1985; Karlin et al., 1994; Woo et al., 2007). Only recently, however, the zinc-finger antiviral protein (ZAP, also known as ARTD13, PARP13, and ZC3HAV1) has been identified as one of the possible driving forces behind the suppression of CpG dinucleotides in vertebrate RNA viruses (Takata et al., 2017). It has been shown that ZAP binds to regions in HIV-1 mRNAs with high CpG content to target them for degradation, thereby reducing viral protein expression and replication (Figure 1, left) (Kmiec et al., 2020; Meagher et al., 2019; Takata et al., 2017). Notably, TRIM25 and KHNYN have been reported as important cofactors since ZAP itself does not degrade viral RNA (Ficarelli et al., 2019; Li et al., 2017; Zheng et al., 2017). KHNYN contains an RNase NYN domain and seems critical for RNA degradation, while the role of TRIM25 in ZAP-mediated restriction is currently less clear. It has been shown that artificial increases in CpG numbers significantly increase the susceptibility of HIV-1 and echoviruses to ZAP inhibition (Ficarelli et al., 2020; Odon et al., 2019; Takata et al., 2017). ZAP shows activity against retro-, alpha-, filo-, hepadna-, picorna-, toga-, herpes-, corona-, and flaviviruses as well as retroelements (Goodier et al., 2015; Nchioua et al., 2020b) and thus, may drive CpG suppression in many viral pathogens. Notably, CpG frequency is not the only determinant of ZAP sensitivity. For example, the number of CpGs at the 5′ end of the *env* gene rather than overall CpG frequency determines ZAP sensitivity of primary HIV-1 strains (Kmiec et al., 2020). The CpG content in mammalian mRNAs varies substantially, and high ZAP levels may even restrict viral RNAs showing degrees of CpG suppression that are similar to those of the human genome (Nchioua et al., 2020b). Most importantly, ZAP also regulates the amounts of hundreds of cellular transcripts. For example, ZAP strongly decreases *TRAILR4* mRNA levels by binding to a region in its 3′ untranslated region (Todorova et al., 2014; Figure 1, right).

In addition to KHNYN, several other host RNases have been shown to degrade viral RNAs. One well-characterized example is RNase L. This nuclease is activated by 2′,5′-oligoadenylates synthesized by oligoadenylate synthetases (OAS) that are induced by IFN and activated by dsRNA (Li et al., 2016; Figure 1, left). Thus, the OAS–RNase L innate immune pathway is specifically induced in the presence of dsRNA and restricts replication of diverse viral pathogens. While dsRNAs are more frequently found in viral RNAs, they also exist in some cellular RNAs, and it has been reported that RNase L degrades both viral and cellular RNAs (Brennan-Laun et al., 2014; Figure 1, right). Intriguingly, knockout of *OAS3* has recently also been shown to rescue replication of viruses with elevated CpG dinucleotide numbers, similar to a knockout of ZAP (Odon et al., 2019). Thus, both factors may target overlapping RNAs. Degradation of retroviral RNAs has also been reported for SAMHD1 (Ryoo et al., 2014; Figure 1), but subsequent findings suggested that this activity is marginal and does not contribute to the antiviral activity of this factor (Antonucci et al., 2016). Notably, antiviral RNases such as RNase L or KHNYN provide an interesting example of antiviral pathways in which target specificity is not determined by the effector itself but by cellular cofactors such as ZAP and OAS that recognize characteristics of viral RNAs. Nevertheless, a sharp distinction of self from non-self RNA is not always possible, and many RNases also cleave various cellular RNAs. It will clearly be of interest to determine which cellular RNAs are affected and to which extent.

At first glance, targeting of self by antiviral factors seems to represent an unintended off-target effect. However, this view may be too simplistic: The regulation of cellular mRNAs by several antivirally active proteins, for example, may actually also be beneficial to the host. This includes ZAP, which may promote apoptosis of cancer cells by depleting *TRAILR4* transcripts (Todorova et al., 2014). Sensing of self RNAs may even boost the potency of anti-cancer drugs. For example, the OAS-RNase L pathway has been shown to enhance the anti-cancer activity of 5-azacytidine since this drug induced the production of cellular dsRNAs (Banerjee et al., 2019).

Antiviral defense factors may not only degrade viral RNAs but also suppress translation of viral proteins without affecting RNA levels. For example, the serine/threonine-protein kinase PKR phosphorylates the eukaryotic translation initiation factor eIF2-α, thereby converting it into a global protein synthesis inhibitor (Dever et al., 1992; Figure 2). Similar to the OAS–RNase L pathway, some specificity is acquired via a dsRNA-dependent activation of PKR. Furthermore, eIF2-α phosphorylation does not necessarily result in a complete shutdown of protein synthesis but allows the translation of specific integrated stress response mRNAs and thus, potentially allows the cell to survive (Pakos-Zebrucka et al., 2016). Notably, however, survival of a cell upon induction of a PKR-mediated stress response requires the simultaneous activation of pro-survival pathways (Qiao et al., 2020).

A more specific discrimination between self and non-self is achieved by IFN-induced proteins with tetratricopeptide repeats (IFITs) (Abbas et al., 2013; Pichlmair et al., 2011). IFIT1 preferentially interacts with tri-phosphorylated RNA (PPP-RNA) that is usually absent in cells from higher eukaryotes but frequently generated during viral replication cycles (Kumar et al., 2014; Figure 1). In contrast to IFIT1, IFIT2 and IFIT3 seem not to interact with viral RNAs but bind to IFIT1 to form the active antiviral complex (Fleith et al., 2018). Altogether, IFITs seem to preferentially target viral as well as misfolded or not properly modified cellular RNAs in the cytoplasm (Gebhardt et al., 2017). IFITs have been shown to suppress translation of viral proteins by interfering with the recruitment of the initiation factor 3 (eIF3) translation complex (Guo et al., 2000; Hui et al., 2003; Figure 2). This suppressive effect may also have detrimental effects on cellular mRNA translation. Many viruses use internal ribosome entry sites (IRES) for cap-independent translation of viral proteins (Martinez-Salas et al., 2017; Roberts and Wieden, 2018), and it has been reported that IFIT1 suppresses IRES-dependent mRNA translation of HCV (Raychoudhuri et al., 2011). While IRES elements are found in many viral genomes, they have also been detected in several cellular RNAs (Godet et al., 2019). Thus, induction of aberrant IFIT expression by IFNs may not only affect the translation of viral proteins but also inhibit the synthesis of specific cellular factors (Figure 1, right). It is well established that all eukaryotic mRNAs contain a 5′ m7G cap (also called cap-0), that is, an N7-methylated guanosine linked to the first nucleotide of the RNA that is critical for proper processing, nuclear export, and cap-dependent protein synthesis (Decroly et al., 2012). Additional methylation at the 2′O position of the initiating nucleotide generates a so-called cap-1. This 2′O methylation allows IFIT proteins as well as the immune sensors RIG-I and MDA5 to discriminate cellular RNAs from others (Ramanathan et al., 2016). IFITs efficiently suppress viral RNAs lacking 2′O methylation in both cell culture and mouse models in an IFN-dependent manner (Abbas et al., 2013; Daffis et al., 2010; Kumar et al., 2014; Pichlmair et al., 2011). Altogether, it is emerging that RNA capping processes are more complex than anticipated, and it will be of interest to further clarify their role in innate antiviral immunity and inflammation.

In comparison to IFITs, another innate immune factor, Schlafen family member 11 (SLFN11), inhibits HIV protein translation in a codon-dependent fashion (Li et al., 2012). Specifically, it has been suggested that SLFN11 exploits the viral codon preference for adenine-rich sequences and sequesters or modifies specific tRNAs to attenuate viral protein synthesis (Figure 2). Notably, epigenetic silencing of SLFN11 expression seems to be associated with resistance to specific cancer drugs (Nogales et al., 2016). The underlying mechanisms remain to be determined, but it has been suggested that epigenetic silencing of SLFN11 might have an impact on the DNA damage response system. Whether or not increased immune activated SLFN11 expression would actually enhance the efficacy of anti-cancer drugs is not known.

### Post-translational modifications of viral proteins

Upon translation, viral proteins depend on a variety of host enzymes that mediate post-translational modifications. These include phosphorylation, N- and O-linked glycosylation, acetylation, the attachment of hydrophobic groups for membrane localization (e.g. myristoylation, GPI anchor addition), and many other processes that determine protein stability, localization, and activity. Consequently, modulation of these modifications may represent an efficient means of the host to interfere with viral replication. One post-translational modification that is targeted by host factors to suppress viral protein maturation is proteolytic cleavage. While many viral pathogens encode proteases to mediate proteolytic processing of their own (poly)proteins, most of them also exploit cellular proteases. One prominent example is the ubiquitously expressed host protease furin/PCSK3 that activates a variety of viral envelope glycoproteins by cleaving a poly-basic consensus motif (R-X-K/R-R↓). Among others, this comprises the envelope (Env) proteins of retroviruses such as HIV-1 (McCune et al., 1988), the hemagglutinin (HA) proteins of highly pathogenic avian influenza A viruses (Kawaoka et al., 1987), the fusion (F) protein of monogenavirales such as human metapneumo- or measles viruses (Richardson et al., 1986), and prM proteins of different flaviviruses (Rice et al., 1985; Stadler et al., 1997). Without proteolytic activation, these viral glycoproteins are not able to mediate fusion of the virion membrane with the target cell. In 2013, Aerts and colleagues identified protease-activated receptor 1 (PAR1) as an endogenous inhibitor of furin (Figure 2) that interferes with the proteolytic activation of the human metapneumovirus F protein (Aerts et al., 2013). In line with the exploitation of furin by many viral pathogens, PAR1 also reduces the processing of the HIV-1 Env precursor gp160 into its mature subunits gp120 and gp41 (Sachan et al., 2019). More recently, guanylate-binding proteins 2 and 5 (GBP2 and GBP5) were also shown to inhibit the enzymatic activity of furin (Figure 2), thereby inhibiting replication of HIV-1, measles virus, Zika virus, and most likely additional furin-dependent viruses (Braun et al., 2019; Krapp et al., 2016). Thus, inhibition of the broadly used virus-dependency factor furin allows the host to restrict replication of diverse viral pathogens. Notably, however, furin also cleaves and activates more than 100 cellular factors, including hormones, growth factors, cytokines, adhesion molecules, and receptors (Braun and Sauter, 2019; Tian et al., 2011). As a result, the expression of PAR1, GBP2, and GBP5 may come at the cost of disturbed host protein maturation. Indeed, increased levels of GBP2 and GBP5 were associated with reduced furin-mediated cleavage of matrix metalloproteinase-14 and glypican-3 (Braun et al., 2019). Although GBP2 and GBP5 are constitutively expressed in many cell types, they belong to the most strongly IFN-γ-inducible proteins. This IFN responsiveness may help to reduce unintended off-target effects and limit expression to cells that are already infected or at risk of infection.

In addition to inhibiting normal post-translation modifications of viral proteins, infected cells may also ‘mark’ viral proteins to prevent them from exerting their functions. For example, ISGylation has been shown to negatively interfere with the stability, activity, and/or assembly of viral proteins (Figure 1, left). This post-translational modification involves the addition of the small ubiquitin-like molecule ISG15 by the HECT and RLD domain containing E3 ubiquitin protein ligase 5 (HERC5). One well-characterized target of HERC5/ISG15 is the non-structural protein 1 (NS1) of IAV. Here, ISGylation abrogates the ability of NS1 to counteract PKR-mediated antiviral effects (Tang et al., 2010). Similarly, ISGylated pUL26 of the human cytomegalovirus loses its ability to suppress NF-κB-mediated immune responses (Kim et al., 2016). The number of viral ISGylation targets is constantly increasing, and accumulating evidence suggests that HERC5/ISG15 do not specifically target individual viral proteins but generally modify newly synthesized proteins (Durfee et al., 2010). In line with this, HERC5/ISG15 is associated with polyribosomes and mediates ISGylation of viral, mammalian, and bacterial substrates in a sequence-independent manner (Durfee et al., 2010). Thus, ISGylation may represent a rather unspecific IFN-induced immune response that does not distinguish between self and non-self (Figure 1). Nevertheless, viruses may be particularly affected due to dominant-negative sterical interference of ISGylated viral proteins with virion assembly. In the case of the L1 capsid protein of human papillomavirus (HPV) and the nucleoprotein (NP) of Influenza B virus, for example, ISGylation inhibits viral particle formation by preventing viral protein assembly (Durfee et al., 2010; Zhao et al., 2016). A similar mechanism has been proposed for HIV-1 Gag (Woods et al., 2011). Negative side effects of ISGylation on the host cell seem highly likely, particularly since attachment of ISG15 also interferes with protein ubiquitination and natural protein turnover (Desai et al., 2006). Nevertheless, these adverse effects may be limited, since only a minor fraction of the total target protein is ISGylated during viral infection (Perng and Lenschow, 2018; Zhao et al., 2016). While this percentage may be sufficient to interfere with virion assembly in a dominant-negative manner, it is tempting to speculate that the function of most cellular proteins may remain largely unaffected. In some cases, ISGylation is also exploited by the host to regulate the activity of cellular factors involved in antiviral immunity. For example, ISG15 enhances antiviral immune responses by stabilizing the transcription factors STAT1 and IRF3 (Malakhova et al., 2003; Shi et al., 2010) and activating PKR (Okumura et al., 2013) but suppresses sensing of viral RNA via ISGylation of RIG-I (Kim et al., 2008; Figure 2).

In summary, these examples illustrate that post-translational modifications represent an effective mechanism of the host to interfere with replication of diverse viral pathogens. However, protein-modifying enzymes frequently affect both viral and cellular proteins, since features generally distinguishing self from non-self proteins are missing. The induction of factors regulating post-translational modifications (e.g. ISG15, GBP2, GBP5) upon viral infection may represent one means to limit potential harmful side effects on the host.

### Discrimination between host and virus membranes during budding

Upon assembly of viral proteins and nucleic acids, enveloped progeny virions bud from cellular membranes. Depending on the virus species, budding takes place in cellular compartments (e.g. ER, Golgi, plasma membrane) or specific virus-induced organelles. Not surprisingly, host factors interfering with membrane composition, transport, or curvature may affect virus budding. For example, the IFN-inducible protein viperin/RSAD2 exerts antiviral activity by interfering with cholesterol metabolism and, thus, lipid composition of membranes. Viperin has been shown to interact with farnesyl diphosphate synthase (FPPS), an enzyme essential for isoprenoid biosynthesis (Figure 2). While one study reported a decrease in the enzymatic activity of FPPS (Wang et al., 2007), a more recent publication demonstrated that viperin decreases total cellular levels of FPPS rather than inhibiting its activity (Makins et al., 2016). As a result of reduced FPPS levels, detergent-resistant membrane microdomains (i.e. lipid rafts) that serve as budding sites for many enveloped viruses do not form properly. A direct link of FPPS depletion and reduced virus release has been demonstrated for IAV (Wang et al., 2007). Whether viperin-mediated restriction of other viral pathogens (e.g. measles virus, CHIKV, HCV, DENV, WNV, HIV) also involves FPPS remains to be determined (Ghosh and Marsh, 2020). Since lipid rafts also serve as platforms for entry of enveloped and non-enveloped viruses, it is tempting to speculate that viperin may additionally interfere with this early step of the viral replication cycle. Intriguingly, viperin also inhibits viral RNA synthesis by converting cytidine triphosphate (CTP) into the chain terminator 3′-deoxy-3′,4′-didehydro-CTP (ddhCTP) (Gizzi et al., 2018). ddhCTP levels are elevated in IFN-α-stimulated cells and inhibit in vivo replication of Zikavirus and potentially other RNA viruses. As a consequence of these independent antiviral activities, viperin may affect the host cell metabolism in several ways: While the production of ddhCTP may suppress cellular transcription, the modulation of FPPS may also come at a cost since lipid rafts play key roles in cellular membrane protein trafficking, signal transduction and receptor trafficking. In line with this, viperin has been shown to reduce cellular protein release (Hinson and Cresswell, 2009; Figure 2).

Another well-characterized and broadly active antiviral factor that targets membranes to inhibit virus release is tetherin/BST-2 (Figure 1; Neil et al., 2008; Van Damme et al., 2008). Instead of altering membrane composition, tetherin acts as a physical leash that prevents the release of newly formed virions from infected cells. This inhibitory activity depends on the unusual topology of tetherin, in which an N-terminal transmembrane domain and a C-terminal GPI anchor are linked by an extracellular coiled-coil domain (Perez-Caballero et al., 2009). The GPI anchor localizes to lipid rafts and is incorporated into the membrane of many enveloped viruses during budding, whereas the transmembrane domain remains attached to the virus-producing cell. This simple, yet effective mechanism allows tetherin to restrict a broad variety of envelope viruses including retro-, filo-, and herpesviruses (Neil, 2013). Furthermore, the localization around lipid rafts, the preferred budding site of many enveloped viruses (Suzuki and Suzuki, 2006), as well as its IFN inducibility may help to limit unwanted side effects on cellular budding events. Nevertheless, it has been shown that tetherin fails to distinguish between budding virions and cellular exosomes as release of the latter is also inhibited (Edgar et al., 2016).

Overall, the current literature suggests that membrane-targeting antiviral factors have the potential to target several steps of the viral replication cycle including fusion, formation of membranous replication complexes, and budding. A clear discrimination between self and non-self membranes can hardly be achieved since viral membranes are always derived from host cell membranes. Nevertheless, some specificity may be conferred by targeting detergent-rich membrane microdomains that serve as entry and budding sites for several viruses or by detecting specific membrane curvatures.

### APOBEC3-induced mutations

Some antiviral factors may exert their inhibitory activity even after successful budding and release of newly formed virions. As discussed above, this includes cellular proteins such as SERINC5 or IFITMs that are incorporated into progeny virions and impair their infectivity. Other cellular factors that are well known to impair virion infectivity, albeit at an even later stage, are members of the APOBEC3 (apolipoprotein B mRNA editing enzyme, catalytic polypeptide-like 3) family. APOBEC3 proteins are cytidine deaminases that interact with viral RNAs and are encapsidated into newly formed virions. They are best established as restriction factors of retroviruses and retrotransposons (Jónsson and Andrésdóttir, 2013). However, they have also been reported to be involved in the control of other RNA viruses (Milewska et al., 2018) as well as some DNA viruses such as herpes-, parvo-, and hepadnaviruses (Janahi and McGarvey, 2013; Nakaya et al., 2016). Their importance is evident from the fact that several virus families evolved APOBEC3 antagonists such as the Vif protein of lentiviruses, the nucleocapsid of HTLV-1, the glyco-Gag of MLV, and the Bet protein of foamy viruses (Harris and Dudley, 2015). In the case of retroviruses, virion incorporation results in deamination of cytosine residues during the reverse transcription process and consequently degradation of reversed transcribed DNA prior to integration as well as lethal G to A coding strand mutations in the integrated provirus. Humans possess seven A3 proteins (A, B, C, D, F, G, and H) resulting from gene duplications on chromosome 22 (Salter et al., 2016). The best characterized antiretroviral factor APOBEC3G preferentially targets CC residues and frequently converts the tryptophan codon TGG to a TAG stop codon (Stavrou and Ross, 2015). Other ABOPEC3 proteins most often target CT motifs and, thus, usually cause GAA or GA to AAA and AA missense mutations, respectively.

However, APOBEC proteins introduce mutations not only in viral nucleic acids but also in cellular nucleic acids. In fact, the first example of mRNA editing observed in vertebrates was the C to U editing of apolipoprotein B (ApoB) mRNA by APOBEC1 (Powell et al., 1987; Teng et al., 1993). This editing step allows the synthesis of two protein isoforms (ApoB48 and ApoB100) from the same precursor mRNA and coined the term ‘APOBEC’. Activation-induced cytidine deaminase (AID), another member of the APOBEC protein family, induces mutations in single-stranded DNA and plays a key role in immunoglobulin diversification (Petersen-Mahrt et al., 2002). While these examples illustrate that editing of viral and cellular nucleic acids may be beneficial to the host, the mutagenic activity of APOBEC proteins may also come at a cost. For example, AID-induced mutations not only increase the antibody repertoire of B cells, but also contribute to the development of B-cell lymphomas (Lenz and Staudt, 2010). Similarly, APOBEC3 proteins, especially APOBEC3B, are emerging as major factors causing mutations in human cancers (Olson et al., 2018; Seplyarskiy et al., 2016; Zou et al., 2017). They may induce C to U deamination of single-stranded cellular DNA that is produced during the repair of double-stranded DNA or becomes accessible on the lagging strand during DNA replication (Petljak et al., 2019; Seplyarskiy et al., 2016). Comprehensive sequence analyses revealed that APOBEC-specific mutation signatures are found in more than half of all human cancer types, albeit with variable impact within each tumor (Alexandrov et al., 2013; Burns et al., 2013; Roberts et al., 2013). Furthermore, increased levels of APOBEC expression due to the presence of high-risk genetic variants or increased IFN-γ signaling are associated with particularly high levels of APOBEC3-mediated mutagenesis in human cancers (Roper et al., 2019). This suggests that chronic inflammation associated with increased IFN levels and expression of APOBEC proteins favors the accumulation of mutations associated with tumor development and metastasis. Altogether, it is becoming evident that APOBEC3 proteins not only protect us against viral pathogens, but also cause somatic mutations driving tumor evolution, metastasis, and/or therapy resistance (Olson et al., 2018). Notably, individual APOBEC3 family members differ in their efficacy against specific RNA and DNA viruses, as well as their contribution to cancer development. Thus, it will be interesting to clarify whether it might be possible to specifically target selected APOBEC3 proteins causing detrimental effects in therapeutic interventions.

### Discrimination between exogenous and endogenous retroviruses

One particular challenge in the discrimination of self from non-self is the recognition of endogenous retroviruses (ERVs) by sensors and effectors of the innate immune response. ERVs are fossils of once infectious retroviruses that make up about 5–8% of the human DNA. Their ancestors infected germ cells and integrated their proviral DNA into the host genome. While many integrated proviruses were lost during evolution, others got fixed in the population and are now inherited in a Mendelian manner. In many cases, these endogenous retroviral sequences are silenced by genetic and epigenetic mechanisms as well as antiviral factors to prevent detrimental effects of their activation and spread. Some ERVs, however, have been co-opted by the host and fulfill important physiological functions in vivo. Consequently, restriction factors targeting retroviral components need to discriminate between beneficial endogenous retroviruses and their harmful counterparts to limit detrimental side effects.

**Figure 3.**
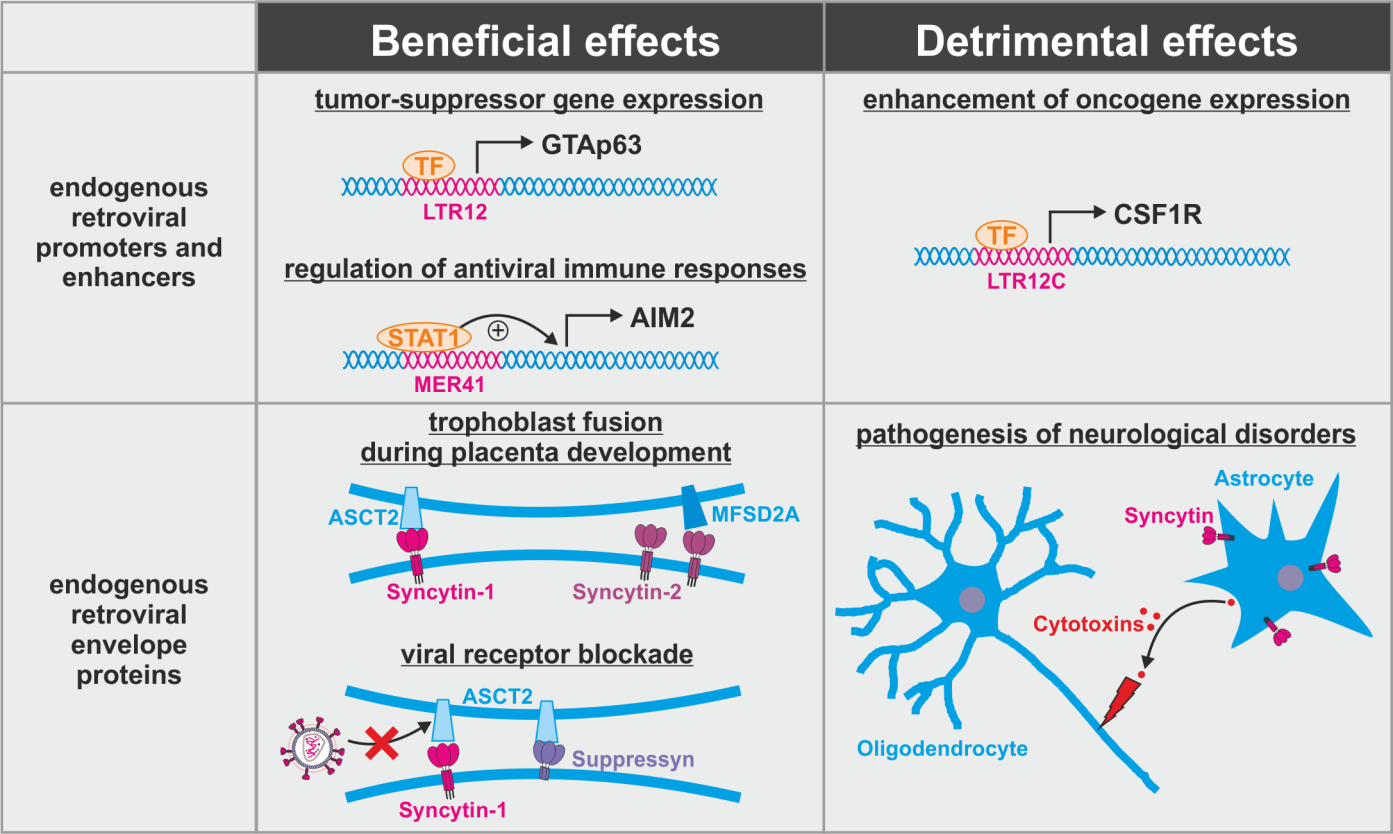
Dual role of endogenous retroviruses (ERVs). ERV-derived regulatory elements (promoters, enhancers, repressors, insulators) and proteins (syncytin-1, syncytin-2, suppressyn, etc.) may have beneficial (left) or detrimental (right) effects on the host. Abbreviations are explained in the text.

One important physiological role of several endogenous retroviruses is their ability to regulate cellular gene expression. ERVs harbor numerous transcription factor binding sites, and many of them act as enhancer or promoter elements for host genes (Cohen et al., 2009; Figure 3, top). For example, expression of the tumor-suppressor GTAp63 is driven by an endogenous retroviral promoter of the LTR12 family (Beyer et al., 2011). Accumulating evidence suggests that *cis*-regulatory ERVs also help to mount an efficient immune response upon infection. Expression of the inflammasome component AIM2, for example, is enhanced by an ERV of the MER41 family (Chuong et al., 2016). Similarly, transcription of *GBP2* and *GBP5* is regulated by endogenous retroviral LTR12C elements (Srinivasachar Badarinarayan et al., 2020). However, aberrant hyperactivation of endogenous retroviral promoters can also enhance the expression of oncogenes such as *CSF1R* (Lamprecht et al., 2010) and contribute to disease progression. Thus, the integration of transposable elements may result in a significant evolutionary conflict. On the one hand, detrimental ERV-derived regulatory elements need to be inactivated by antiviral factors such as KAP1 that epigenetically silences transposable elements (Ecco et al., 2017). On the other hand, ERV promoters and enhancers that provide a selection advantage need to be excluded from these silencing mechanisms. Aberrant ERV-driven expression of oncogenes such as *CSF1R* or *IRF5* in cancer cells illustrates that this discrimination is not always successful and may lead to severe disease (Babaian and Mager, 2016).

The evolutionary conflict associated with the fixation and co-option of ERVs is further illustrated by the exaptation of ERV-derived Env proteins such as syncytin-1 or syncytin-2 (Figure 3, bottom). These two envelope proteins have retained their activity upon fixation in humans where they mediate the fusion of trophoblast cells into the syncytiotrophoblast, an essential step during placenta formation (Blaise et al., 2003; Mi et al., 2000). This fusion step closely resembles the fusion of viral and cellular membranes mediated by the envelope proteins of pathogenic exogenous retroviruses. Not surprisingly, antiviral host proteins targeting retroviral fusion events fail to distinguish between beneficial and detrimental retroviral Env proteins. As already noted above, this includes IFITM1-3 that have been shown to suppress syncytin-mediated trophoblast fusion if expressed in the placenta (Buchrieser et al., 2019). Most likely, other factors targeting retroviral membrane fusion (e.g. SERINCs, CH25H) or Env maturation (e.g. PAR1, GBP2, GBP5) may result in similar unwanted side effects if expressed in the placenta. Another retroviral Env protein that has been co-opted during primate evolution is suppressyn that fails to mediate fusion as it lacks parts of its C-terminal domain (Sugimoto et al., 2013). Nevertheless, it may act as important regulator of placenta formation since it shares its receptor ASCT2 with syncytin-1 (Sugimoto et al., 2013). Furthermore, blockage of ASCT2 by suppressyn has recently been suggested to protect primates from infection with RD114/simian type D retroviruses that use the same receptor for entry (Frank et al., 2020). Whether or how suppressyn activity is affected by antiretroviral host proteins remains unclear. Finally, some of the co-opted ERV-derived envelope proteins may contribute to pathogenesis of neurological disorders (Dolei et al., 2015). This includes the induction of neuroinflammation and oligodendrocyte death by syncytin-mediated release of cytotoxins by astrocytes (Antony et al., 2007). Thus, ERV-derived proteins cannot be simply categorized into good and evil, and antiviral host proteins targeting ERVs may have beneficial or detrimental effects depending on their level, timing, and site of expression.

### Long-term effects of antiviral factors on host evolution

**Figure 4.**
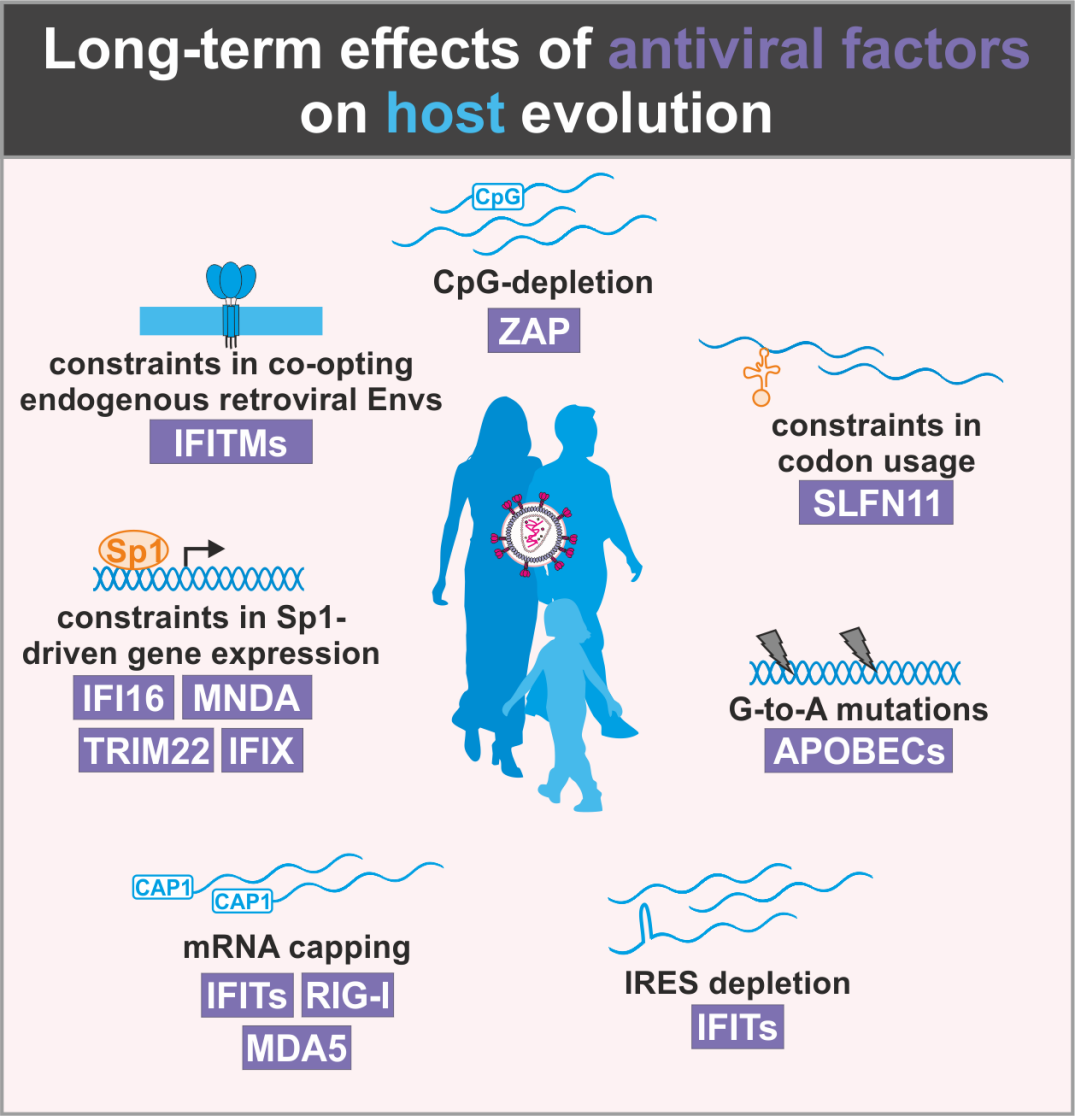
Long-term effects of antiviral proteins on host evolution. Antiviral proteins (violet) exert selection pressure on host factors to limit similarities with viral factors. As a result, the emergence of antiviral cellular factors may be associated with constraints in host evolution.

Importantly, the ever-ongoing battle with viral pathogens has not only consequences for the individuum but also created and still shapes most parts of the human genome. This is most obvious from the fact that more than half of the human genome is composed of transposable elements (e.g. LINEs, SINEs, HERV), while only 1–2% encode for proteins (Dunham et al., 2012). Furthermore, human evolution is under numerous constraints in order to maintain effective innate antiviral defense mechanisms while avoiding severe adverse effects (Figure 4). For example, the human genome must maintain low levels of CpG dinucleotides and has to avoid utilization of specific codons to prevent cellular mRNA degradation or suppression of translation by ZAP and SLFN11, respectively. While APOBEC3 proteins preferentially target single-stranded viral RNAs and DNAs, they also introduce mutations in the human genome (Pinto et al., 2016) and play a key role in cancer development (Seplyarskiy et al., 2016). Thus, the human genome is under selection pressure for suppression of APOBEC3 recognition motifs and may accumulate APOBEC3-induced mutations over time. Similarly, mRNA secondary structures such as IRES elements or mRNAs without 5′ cap are under negative selection as they may be targeted by different sensors and effectors of antiviral immunity. Accumulation evidence shows that several IFN-inducible factors restrict viral gene expression by limiting the availability of the transcription factor Sp1. This factor is also involved in many cellular processes such as differentiation, growth, apoptosis, immune, and DNA responses as well as chromatin remodeling. It is conceivable, however, that a transcription factor that becomes limiting under conditions of infection and/or inflammation should not become too important to ensure proper functioning of the cell and the organisms under these conditions. Finally, the co-option of endogenous retroviral Gag or Env proteins by the host cell is complicated by the presence of antiviral factors targeting exactly these structures. The exploitation of Env-derived syncytins provides a prime example as they are essential for placenta development in humans but may be inhibited by IFITMs.

### Conclusion and perspectives

It is tempting to speculate how the human genome might have evolved in the absence of antiviral factors. Most likely, humans would have benefitted from a larger flexibility in the primary sequence, secondary structure, and modification of mRNAs due to the lack of RNA-binding antiviral proteins. In this case, the lack of constraints may have facilitated the evolution of novel mechanisms regulating gene expression and translation as well as a faster adaptation of host genes to novel selection pressures. Moreover, the absence of antiviral factors targeting epigenetic modifications, transcription or translation may have allowed a larger flexibility in the tissue- and cell type-specific expression of cellular genes and facilitated the evolution of new transcript variants and protein isoforms. Apart from gene expression and protein synthesis, membrane budding and fusion events within and between cells may have evolved in a different manner since any similarities with viral entry or budding events would not be problematic. On the other hand, however, the absence of viruses and antiviral factors would have precluded the integration and exploitation of (retro)viral sequences. This includes the co-option of virus-derived *cis*-regulatory elements (e.g. promoters, enhancers, insulators) as well as viral proteins (e.g. Env). Notably, the presence of repetitive viral elements also facilitates gene loss and duplication events and, thus, faster adaptation of the human organism to an ever-changing environment. Similarly, the mutagenic activity of antiviral factor such as APOBEC3 proteins may not always have detrimental effects but also facilitate adaptation of the host to environmental changes. Thus, targeting of cellular nucleic acids or proteins by antiviral factors may not necessarily be detrimental, but also help the host to regulate cellular processes, particularly in response to stress stimuli that induce the expression of antiviral proteins. Consequently, both the absence and presence of antiviral factors as well as endogenous viral elements may provide selection advantages to the host. At the end of the day, host organisms may have found a balance that allows them to efficiently fight off most of the viral pathogens they encounter, while tolerating a few drawbacks that may be associated with the activity of antiviral proteins. One interesting question is whether special features may allow some species to minimize adverse effects of innate immune mechanisms. For example, it has been suggested that the high body temperatures and metabolic rates achieved during flight promoted the evolution of reduced reaction to foreign and self-DNAs in bats (Banerjee et al., 2020). Without this adaptation, the DNA damage that is associated with high metabolic activity would most likely result in detrimentally increased sensing of self-DNA. Since other vertebrate species also differ in their body temperatures and metabolic activities, it will be interesting whether such protective mechanisms are confirmed, for example, in birds. Metabolic activities may even exert protective effects in human individuals since anti-inflammatory effects of physical exercise are well documented although the underlying mechanisms remain poorly understood (Nieman and Wentz, 2019).

While we can only speculate about how humans may have evolved in a world without viruses, there is one thing we can say for certain: the human organism has been shaped to a large extent by viruses. This is not only due to the presence of hundreds of thousands of endogenous retroviral sequences in our genome but also due to the consequence of the evolution of antiviral factors that have driven the evolution of the entire human genome.
